# Pretreatment systemic immune-inflammation index and lymphocyte-to-monocyte ratio as prognostic factors in oral cavity cancer: A meta-analysis

**DOI:** 10.1097/MD.0000000000040182

**Published:** 2024-11-01

**Authors:** Jianghan Xu, Yanjun Lin, Jingbo Yang, Yifeng Xing, Xiaojie Xing

**Affiliations:** a Key Laboratory of Oral Diseases and Fujian Provincial Engineering Research Center of Oral Biomaterial and Stomatological Key Lab of Fujian College and University, School and Hospital of Stomatology, Fujian Medical University, Fuzhou, China; b Department of Medical Oncology, The Second Affiliated Hospital of Fujian Medical University, Quanzhou, China.

**Keywords:** lymphocyte-to-monocyte 2 3 ratio, meta-analysis, oral cavity cancer, prognosis, systemic immune-inflammation index

## Abstract

**Background:**

The predictive implications of the pretreatment systemic immune-inflammation index (SII) and lymphocyte-to-monocyte ratio (LMR) in oral cavity cancer have been investigated extensively, however, the findings are conflicting.

**Methods:**

To assess the predictive importance of SII and LMR in patients with oral cavity cancer, a comprehensive Meta-analysis of the literature was conducted using the databases from PubMed, Embase, and the Cochrane Library. To determine the link between SII and LMR and overall survival (OS) and disease-free survival (DFS), hazard ratio (HR) and 95% confidence interval (CI) were retrieved.

**Results:**

The analysis comprised a total of 18 papers, covering 19 trials (SII = 5, LMR = 12, SII + prognostic nutritional index (PNI) = 2). According to pooled data, increased SII predicted poor OS (HR: 1.61, 95% CI: 1.38–1.87, *P* < .001) and DFS (HR: 1.90, 95% CI: 1.11–3.27, *P* = .02) while high LMR was linked with improved OS (HR: 0.64, 95% CI: 0.54–0.77, *P* < .001) and DFS (HR: 0.69, 95% CI: 0.61–0.79, *P* < .001). In addition, subgroup analysis indicated that high SII and low LMR negatively correlated with OS regardless of country, cutoff value, sample size, or types of Cox regression analysis.

**Conclusions:**

High SII and low LMR may predict worse survival in patients with oral cavity cancer. SII and LMR may therefore represent effective indicators of prognosis in oral cavity cancer.

## 1. Introduction

The most frequent kind of cancer in the head and neck region, oral cavity cancer, is one of the main causes of cancer fatalities and a serious public health concern globally.^[1,2]^ Oral cavity cancer is expected to be the eighteenth most often diagnosed cancer and the sixteenth main cause of cancer-related mortality globally, with more than 370,000 new cases and 170,000 deaths per year, according to global cancer data.^[3]^ Despite advances in illness detection, staging, and therapy, 5-year overall survival (OS) has not grown appreciably during the last 2 decades.^[4]^ Surgery, radiation, and chemotherapy have been the primary methods for managing oral cavity cancer, either individually or in combination, thus far. Additionally, each patient requires personalized treatment strategies based on factors like cancer type, stage, presence of metastasis or lymph node involvement, and overall health. Typically, early-stage cancers are treated with a single method such as surgery or radiotherapy, while advanced cases often require a combination of treatments like surgery with chemoradiotherapy or chemotherapy with immunotherapy to improve prognosis. Designing a tailored treatment plan based on patients’ predicted survival time can be advantageous in improving the cure rate of oral cavity cancer and enhancing the quality of patient survival. Currently, the tumor-node-metastasis staging approach is extensively used to estimate survival in patients with oral cavity cancer.^[5]^ Despite this, individuals with equal tumor-node-metastasis stages frequently have varying outcomes despite getting similar therapy. As a result, finding novel prognostic biomarkers to predict therapy response or long-term survival in patients with oral cavity cancer is critical.

It has been demonstrated that host systemic inflammatory responses play a key role in carcinogenesis and disease development.^[6]^ Meta-analyses of the predictive usefulness of peripheral blood inflammation-based markers such as neutrophil-to-lymphocyte ratio^[7,8]^ and platelet-to-lymphocyte ratio^[9]^ in oral cavity cancer have recently sparked great attention. Systemic immune-inflammation index (SII), a novel integrated score calculated by multiplying the platelet count by the neutrophil count then dividing by the lymphocyte count, is used to predict poor prognosis in cancers of the gastrointestinal tract,^[10]^ urinary tract,^[11,12]^ gynecology^[13]^ and lung.^[14,15]^ However, the use of SII to indicate prognosis in patients with oral cavity cancer remains inconsistent. Furthermore, SII, which is based on the counts of 3 different types of circulating immune cells, may more accurately reflect the balance of host immunological and inflammatory state than neutrophil-to-lymphocyte ratio and platelet-to-lymphocyte ratio, which are based on just 2 types. The SII represents more robust indicators of prognosis in oral cavity cancer than other inflammation-based markers. Similarly, another inflammation-based biomarker, lymphocyte-to-monocyte ratio (LMR), based on peripheral blood lymphocyte and monocyte count, has been observed as an independent prognostic factor for lung cancer,^[16]^ gastrointestinal cancers,^[17]^ urinary system cancers,^[18,19]^ and gynecological cancers,^[20]^ but its use in oral cavity cancer remains uncertain. We therefore performed a meta-analysis to evaluate the predictive impact of SII and LMR in patients with oral cavity cancer.

## 2. Methods

### 2.1. Selection criteria and search approach

The PubMed, Embase, and Cochrane Library databases were looked through for relevant papers published up to June 2023 that examined the association between SII or LMR and oral cavity cancer’s prognosis. “Systemic immune-inflammation index,” “SII,” “oral cavity cancer,” “oral squamous cell carcinoma,” “lymphocyte-to-monocyte ratio,” “LMR,” “prognosis,” and “prognostic” were the search keywords utilized. To locate more relevant research, references referenced in chosen papers were also collected.

All candidate articles were evaluated by 2 independent writers. Studies were deemed qualified if they satisfied the following requirements: included patients with histopathologically confirmed oral cavity cancer; reported hazard ratios (HRs) and 95% confidence intervals (CIs) for OS, or disease-free survival (DFS); or included sufficient data to calculate HR and 95% CI; and published in English. Among the exclusion criteria were: letters, reviews, and case reports; HR and 95% CI were not supplied or could not be computed; the cutoff value was not provided; and studies were not published in English.

### 2.2. Data extraction and quality evaluation

Two authors thoroughly considered the eligible studies to acquire the following details: the surname of the first author, the country of origin, the year of publication, the number of patients, the study design, the patient’s age, the cutoff value, the duration of follow-up, and the survival analysis. Each study’s HR from multivariate or univariate analysis was retrieved to produce a summary HR with 95% CI. Three investigators individually assessed the quality of all primary studies using the Newcastle–Ottawa Quality Assessment Scale (NOS).^[21]^ NOS ratings of 6 are considered high-quality research, with a maximum value of 9.

### 2.3. Statistical investigation

HR and 95% CI data were gathered directly from individual papers or computed as previously stated^[22]^ if not presented directly. We calculated the pooled HRs from each study in multivariate models where available. We used Cochran Q and *I*^2^ statistical methods to assess the statistical heterogeneity of combined results.^[23]^ If *I*^2^ was less than 50% or the *P* value was greater than .10, we considered our findings to be not influenced by heterogeneity. In such cases, the combined results were obtained using a fixed-effects model, while a random-effects model was employed as an alternative. Subgroup analysis was also performed based on nation, sample size, cutoff value, and Cox regression analysis method. Egger’s test and a funnel plot were used to assess potential publication bias. A statistically significant *P* value of .05 was used. Furthermore, a sensitivity analysis was performed by successively eliminating each study to analyze the impact of each individual study on the aggregate results. Stata software version 12.0 was used for all analyses.

## 3. Results

### 3.1. Characteristics of chosen articles

**Figure 1.**
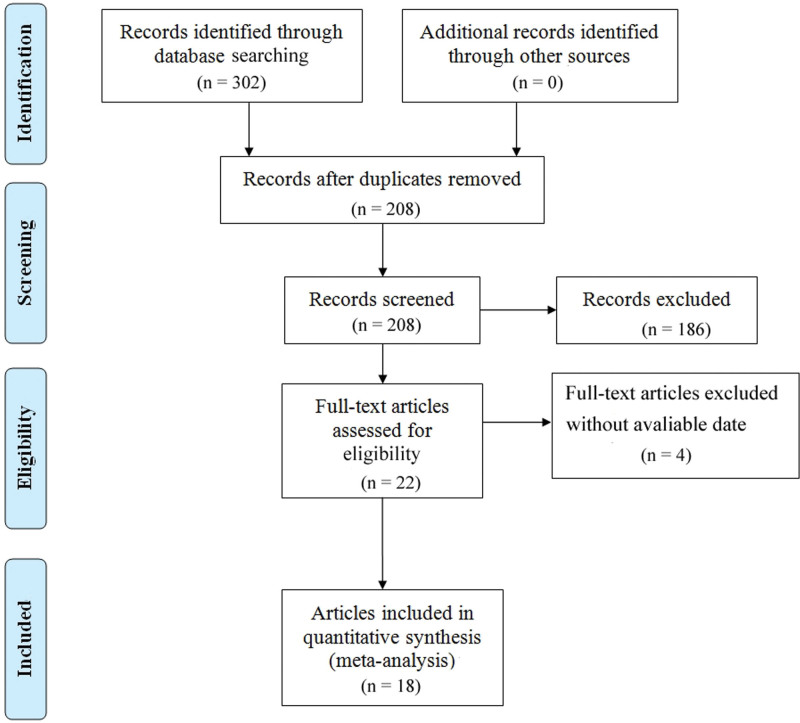
Diagram showing the meta-analysis process.

Figure 1 depicts a flow chart of the selection method. The database search yielded 302 possibly relevant papers, of which 280 were eliminated after examining titles and abstracts. Four publications were removed after further screening of the entire text of the remaining 22 research due to a lack of accessible data. Finally, 18 articles with 19 studies representing a total of 6571 patients were selected for meta-analysis.^[24–41]^ Table 1 shows the features of the 19 studies. Five studies investigated SII, 12 investigated LMR, and 2 investigated both SII and LMR. According to the NOS, all the included studies were of good quality. Twelve of the investigations were done in China, 5 in Japan, and 2 in Spain or Korea. The sample sizes varied from 101 to 993 patients, with a median follow-up period of 31 to 105.6 months. Eight of the included studies assessed OS, whereas 11 assessed both OS and DFS.

**Table 1:** Characteristics of studies included in this meta-analysis.

Study, year	Country	Duration	Study design	Sample size	Age	Gender (M/F)	Follow-up (mo)	Prognostic markers	Survival outcome	Cutoff	Survival analysis	NOS
Ong 2017	China	2009–2013	R	133	median: 51.92	71/62	median: 52	LMR	OS/DFS	5.3/3.2	M	8
Diao 2018 1	China	2006–2016	R	138	NR	81/57	median: 48	SII	OS/DFS	484.5	M	8
Diao 2018 2	China	2004–2014	R	171	NR	90/81	median: 45	SII	OS/DFS	484.5	M	8
Eltohami 2018	China	2005–2014	R	613	mean: 50.5	556/57	NR	LMR	OS/DFS	4.85	U	6
Furukawa 2019	Japan	2001–2015	R	103	median: 63	55/48	NR	LMR	OS	4.29	M	7
Jariod-Ferrer 2019	Spain	2011–2014	R	215	median: 67.5	145/70	median: 31	LMR	OS	2.6	M	8
Watabe 2019	Japan	2004–2012	R	110	median: 68	61/49	median: 68	LMR	OS/DFS	5	M	8
Hasegawa 2020	Japan	2001–2013	R	433	mean: 66.3	246/187	mean: 59.1	LMR	OS	4.35	U	7
Hung 2020	China	2005–2012	R	993	median: 51	922/71	median: 105.6	SII	OS	810.6	M	8
Lee 2020	Korea	2005–2018	R	291	median: 63	108/183	mean: 41	LMR	OS/DFS	4.65/4.45	U	7
Lu 2020	China	2012–2017	R	120	median: 55	79/41	median: 37.5	LMR, SII	OS/DFS	4.02, 569	U, M	8
Mikoshiba 2020	Japan	1991–2018	R	101	median: 59	63/38	median: 65	LMR	OS/DFS	5.54	M	8
Chen 2021	China	2010–2017	R	651	NR	423/228	median: 31.1	LMR	OS	3.18	M	7
Ding 2021	China	2012–2015	R	493	NR	261/232	NR	LMR	OS/DFS	3.4	M	7
Lin 2021	China	2008–2019	R	169	median: 57	93/76	median: 50	LMR	OS	4.15	M	8
Wei 2021	China	2008–2019	R	172	median: 69	96/76	NR	LMR, SII	OS	3.4, 20.4	U	6
Wu 2021	China	2005–2012	R	890	median: 50.8	828/62	median: 72.7	LMR	OS	4.21	U	7
Huang 2022	China	2011–2020	R	592	mean: 54.2	518/74	median: 100	SII	OS/DFS	337.1/348.33	M	8
Kubota 2022	Japan	2005–2017	R	183	median: 66	103/80	NR	SII	OS/DFS	569	U/M	7

DFS = disease-free survival, M = multivariate, NOS = Newcastle-Ottawa Quality Assessment Scale, NR = not reported, OS = overall survival, R = retrospective, U = univariate.

### 3.2. SII’s predictive value in oral cavity cancer

**Figure 2.**
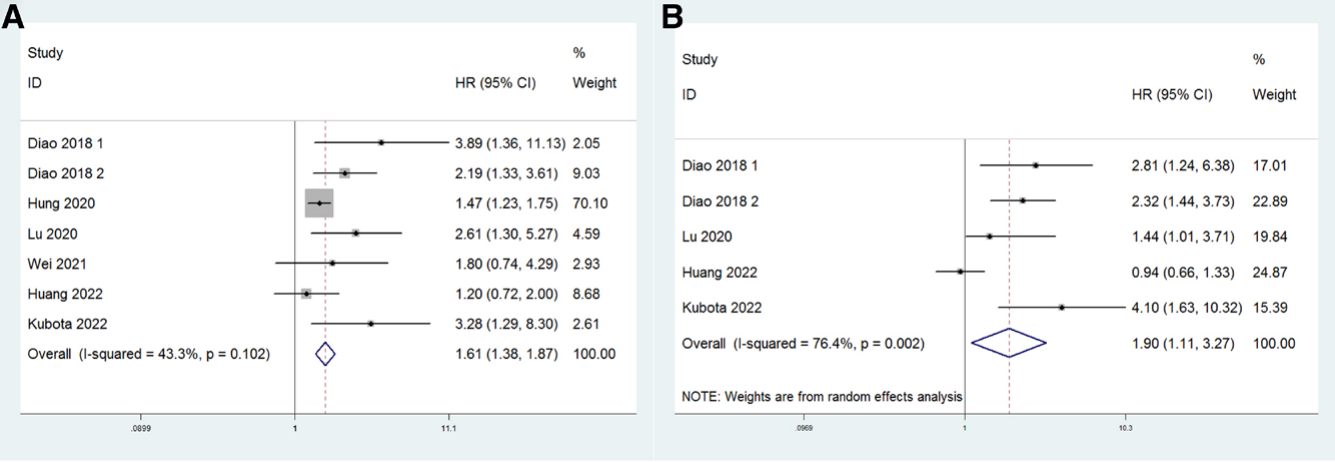
Forest plot of the relationship between high SII and OS or DFS. DFS = disease-free survival, OS = overall survival, SII = systemic immune-inflammation index.

Seven research investigated the impact of SII on OS. Patients with high SII had a shorter life expectancy than those with low SII (HR: 1.61, 95% CI: 1.38–1.87, *P* < .001). Because there was no significant heterogeneity (*I*^2^ = 43.3%, *P* = .102), the fixed-effect model was employed for analysis (Fig. 2A). Subgroup analysis revealed that high SII predicted poor overall survival in Chinese patients (HR: 1.58, 95% CI: 1.35–1.84, *P* < .001). After grouping by sample size, the pooled HR for sample size > 200 was 1.43 (95% CI: 1.21–1.70, *P* < .001) and 2.46 (95% CI: 1.77–3.41, *P* < .001) for sample size < 200. Moreover, when SII > 484.5, pooled HR for OS was 2.03 (95% CI: 1.20–3.44, *P* = .009) and when SII ≤ 484.5, pooled HR for OS was 1.79 (95% CI: 1.30–2.45, *P* < .001). In the analysis of type of Cox regression method, pooled HR for patients from multivariate analysis was 1.78 (95% CI: 1.30–2.45, *P* < .001) and 2.39 (95% CI: 1.26–4.53, *P* = .008) from univariate analysis. These results are shown in Table 2.

**Table 2:** Subgroup analyses of SII.

Subgroup	No. of studies	HR (95% CI)	*P*	Heterogeneity		Model
				*I*^2^ (%)	Ph	
Overall survival						
Country						
China	6	1.58 (1.35–1.84)	<.001	39.6	0.142	Fixed
Japan	1	3.28 (1.29–8.32)	.012	–	–	–
Sample size						
＞200	2	1.43 (1.21–1.70)	<.001	0	0.469	Fixed
<200	5	2.46 (1.77–3.41)	<.001	0	0.769	Fixed
Cut-off						
＞484.5	3	2.03 (1.20–3.44)	.009	59.8	0.083	Random
≤484.5	4	1.79 (1.30–2.45)	<.001	40.7	0.167	Fixed
Analysis						
Multivariate	5	1.78 (1.30–2.45)	<.001	51.1	0.085	Random
Univariate	2	2.39 (1.26–4.53)	.008	0	0.358	Fixed
Disease-free survival						
Country						
China	4	1.64 (0.95–2.82)	.073	75.3	0.007	Random
Japan	1	4.10 (1.63–10.32)	.003	–	–	–
Sample size						
＞200	1	0.94 (0.66–1.33)	.729	–	–	–
<200	4	2.28 (1.65–3.15)	<.001	19.7	0.291	Fixed
Cut-off						
＞484.5	2	2.30 (0.83–6.39)	.109	69.7	0.069	Random
≤484.5	3	1.74 (0.84–3.62)	.138	83.5	0.002	Random
Analysis						
Multivariate	5	1.90 (1.11–3.27)	.02	76.4	0.002	Random
Univariate	0	–	–	–	–	–

HR = hazard ratio, SII = systemic immune-inflammation index.

Five studies examined the effect of SII on DFS. Patients with high SII had poorer DFS than those with low SII, according to the pooled data (HR: 1.90, 95% CI: 1.11–3.27, *P* = .02). Huge heterogeneity (*I*^2^ = 76.4%, *P* = .002) was found, and the random model was therefore used for analysis (Fig. 2B). Subgroup studies revealed that increased SII was unrelated to DFS in Chinese patients (HR: 1.64, 95% CI: 0.95–2.82, *P* = .073). After stratification by sample size, the pooled HR was 0.94 (95% CI: 0.66–1.33, *P* = .729) for sample size > 200 and 2.28 (95% CI: 1.65–3.15, *P* < .001) for sample size < 200. Moreover, when SII > 484.5, pooled HR for DFS was 2.30 (95% CI: 0.83–6.39, *P* = .109) and when SII ≤ 484.5, pooled HR for DFS was 1.74 (95% CI: 0.84–3.62, *P* = .138). All the pooled HR evaluated DFS were from multivariate analysis, the subgroup based on type of Cox regression method therefore not conducted. These results are also shown in Table 2.

### 3.3. LMR’s prognostic value in oral cavity cancer

**Figure 3.**
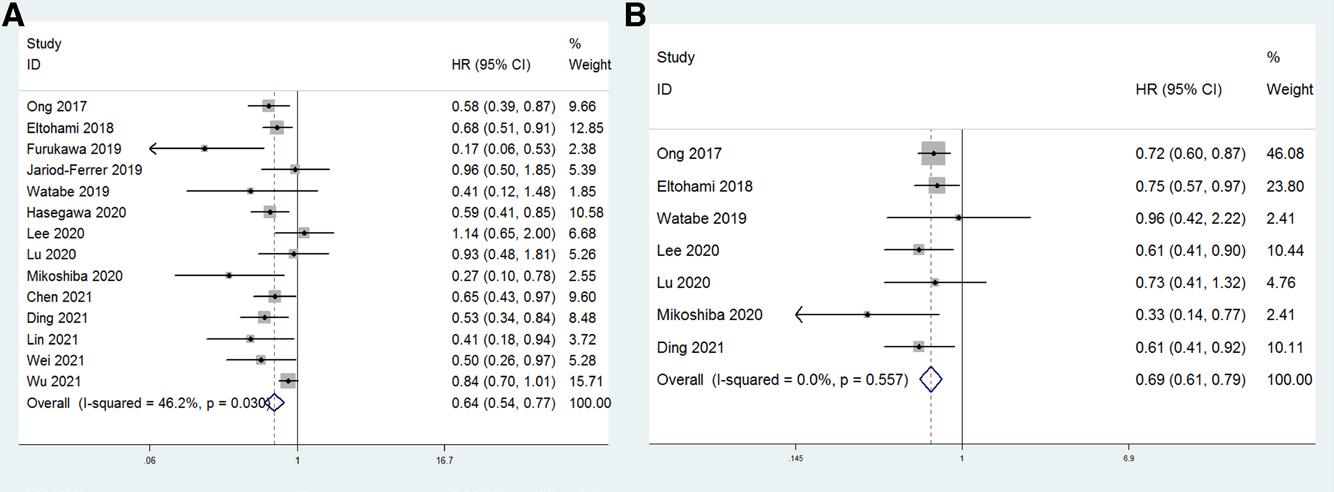
Forest plots of studies evaluating the association between high LMR and OS or DFS. DFS = disease-free survival, LMR = lymphocyte-to-monocyte ratio, OS = overall survival.

Combined data suggested that increased LMR significantly correlated with good OS, with a pooled HR estimate of 0.64 (95% CI: 0.54–0.77, *P* < .001; Fig. 3A) with moderate heterogeneity (*I*^2^ = 46.2%, *P* = .030). Subgroup analyses showed that increased LMR predicted better OS among patients in China (HR: 0.71, 95% CI: 0.63–0.81, *P* < .001) and non-China countries (HR: 0.56, 95% CI: 0.34–092, *P* = .023). Following stratification by sample size, pooled HR was 0.75 (95% CI: 0.66–0.85, *P* < .001) for sample size > 200 and 0.52 (95% CI: 0.40–0.67, *P* < .001) for sample size < 200. Moreover, when LMR > 4.21, pooled HR for OS was 0.58 (95% CI: 0.43–0.79, *P* = .001) and when LMR ≤ 4.21, pooled HR for OS was 0.75 (95% CI: 0.65–0.87, *P* < .001). In addition, no matter pooled the multivariate or univariate Cox regression results, high LMR had significant prognostic value in patients (Multivariate: HR: 0.56, 95% CI: 0.45–0.68, *P* < .001; Univariate: HR: 0.77, 95% CI: 0.67–0.88, *P* < .001). These results are shown in Table 3.

**Table 3:** Subgroup analyses of LMR.

Subgroup	No. of studies	HR (95% CI)	*P*	Heterogeneity		Model
				*I*^2^ (%)	Ph	
Overall survival						
Country						
China	8	0.71 (0.63–0.81)	<.001	23.9	0.239	Fixed
Non-China	6	0.56 (0.34–0.92)	.023	65.4	0.013	Random
Sample size						
＞200	7	0.75 (0.66–0.85)	<.001	31	0.192	Fixed
<200	7	0.52 (0.40–0.67)	<.001	34.7	0.163	Fixed
Cut-off						
＞4.21	7	0.58 (0.43–0.79)	.001	55	0.038	Random
≤4.21	7	0.75 (0.65–0.87)	<.001	28.9	0.208	Fixed
Analysis						
Multivariate	8	0.56 (0.45–0.68)	<.001	32.8	0.166	Fixed
Univariate	6	0.77 (0.67–0.88)	<.001	30.9	0.204	Fixed
Disease-free survival						
Country						
China	4	0.71 (0.62–0.82)	<.001	0	0.869	Fixed
Non-China	3	0.59 (0.43–0.83)	.002	36.4	0.208	Fixed
Sample size						
＞200	3	0.68 (0.56–0.82)	<.001	0	0.581	Fixed
<200	4	0.71 (0.59–0.84)	<.001	19.3	0.294	Fixed
Cut-off						
＞4.21	4	0.68 (0.56–0.84)	<.001	30	0.232	Fixed
≤4.21	3	0.70 (0.59–0.83)	<.001	0	0.753	Fixed
Analysis						
Multivariate	4	0.69 (0.58–0.81)	<.001	27.1	0.249	Fixed
Univariate	3	0.70 (0.57–0.87)	.001	0	0.687	Fixed

HR = hazard ratio, LMR = lymphocyte-to-monocyte ratio.

Similarly, high LMR indicated greater DFS, with a pooled HR estimate of 0.69 (95% CI: 0.61–0.79, *P* < .001; Fig. 3B) with no heterogeneity (*I*^2^ = 0.0%, *P* = .557). As shown in Table 3, subgroup analyses showed that increased LMR predicted better DFS among patients in China (HR: 0.71, 95% CI: 0.62–0.82, *P* < .001) and non-China countries (HR: 0.59, 95% CI: 0.43–0.83, *P* = .002). Following stratification by sample size, pooled HR was 0.68 (95% CI: 0.56–0.82, *P* < .001) for sample size > 200 and 0.71 (95% CI: 0.59–0.84, *P* < .001) for sample size < 200. Moreover, when LMR > 4.21, pooled HR for DFS was 0.68 (95% CI: 0.56–0.84, *P* < .001) and when PNI ≤ 4.21, pooled HR for DFS was 0.70 (95% CI: 0.59–0.83, *P* < .001). In the analysis of type of Cox regression method, pooled HR for patients from multivariate analysis was 0.69 (95% CI: 0.58–0.81, *P* < .001) and 0.70 (95% CI: 0.57–0.87, *P* = .001) from univariate analysis.

### 3.4. Analysis of sensitivity and publication bias

**Figure 4.**
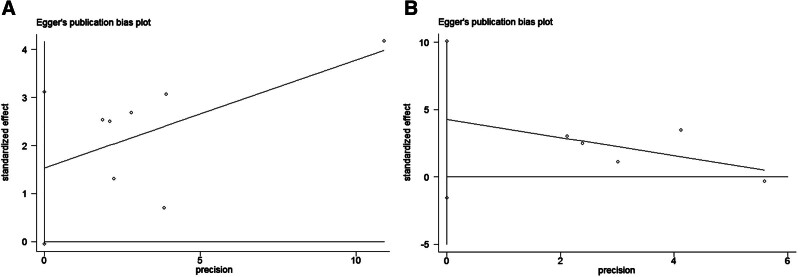
Publication bias funnel plots by Egger. High SII’s relationship to OS (A), and DFS (B). DFS = disease-free survival, OS = overall survival, SII = systemic immune-inflammation index.

**Figure 5.**
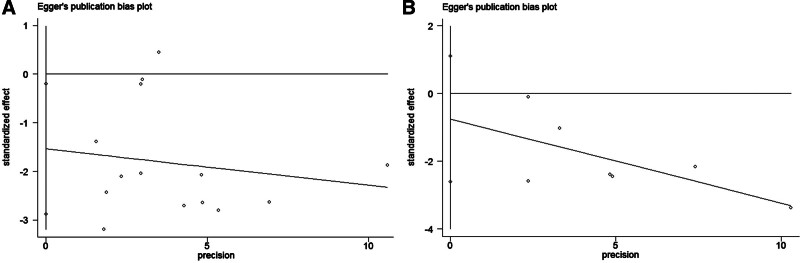
Publication bias funnel plots by Egger. High LMR’s relationship to OS (A), and DFS (B). DFS = disease-free survival, LMR = lymphocyte-to-monocyte ratio, OS = overall survival.

**Figure 6.**
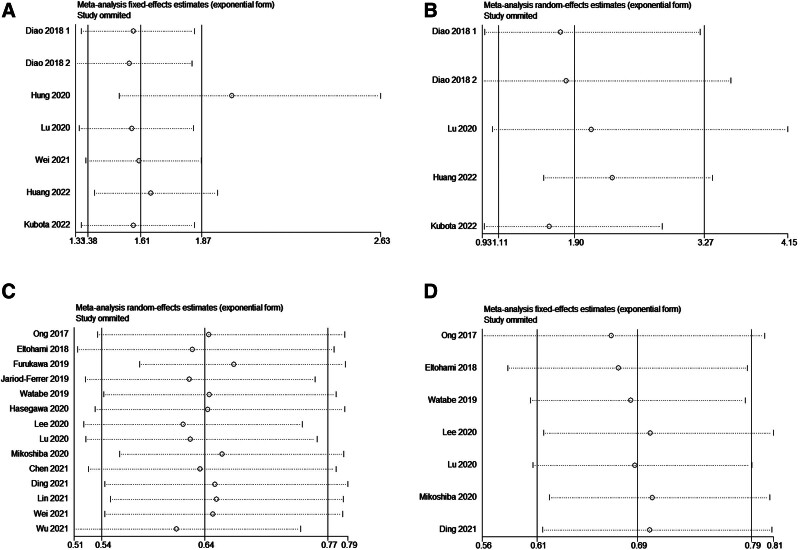
Analyzing the meta-analysis’s sensitivity. High SII correlates with OS (A) and DFS (B); high LMR correlates with OS (C) and DFS (D). DFS = disease-free survival, LMR = lymphocyte-to-monocyte ratio, OS = overall survival, SII = systemic immune-inflammation index.

As shown in Figure 4A and B, publication bias was not significant in studies on SII and pooled OS (*P* = .055) or DFS (*P* = .102) based on Egger’s test and funnel plots. In addition, publication bias was existed in studies on LMR and pooled OS (*P* = .028; Fig. 5A) but not significant in studies on LMR and pooled DFS (*P* = .346; Fig. 5B). The possible influence of published studies on the pooled data was estimated using sensitivity analysis. As demonstrated in Figure 6, the pooled HR did not change appreciably when each study was eliminated in turn.

## 4. Discussion

This meta-analysis of 19 studies in 6571 patients investigated the relationship between SII and LMR with prognosis in patients with oral cavity cancer. Our results indicated that pretreatment SII and LMR may represent independent prognostic indicators in this patient population. Increased SII was associated with poor OS and DFS, and increased LMR was significantly correlated with better OS and DFS. A subgroup analysis demonstrated that high LMR were significantly correlated with increased OS or DFS in patients with oral cavity cancer, irrespective of the country, cutoff value of the LMR, sample size and the methods of survival analysis. In addition, the negative effect of high SII on the OS was detected in every subgroup with varying the country, sample size, cutoff value and methods of survival analysis. Due to the insufficient included studies, we did not find a correlation between SII and DFS in some subgroups. Further randomized controlled studies (RCTs) with large sample size were needed to discover their correlation.

The link between inflammation and cancer has received a lot of attention.^[42]^ Tumour cells secrete pro-inflammatory chemicals, and systemic inflammation enhances tumor cell proliferation, migration, and invasion by blocking apoptosis and increasing angiogenesis.^[43,44]^ SII, a marker of systemic inflammation, is calculated as platelet count × neutrophil count/lymphocyte count. Patients who have a high SII score often exhibit elevated levels of thrombocytosis, neutrophilia, or lymphopenia. Tumor-associated platelets play a role in promoting tumor growth and invasion by releasing growth factors and chemical molecules like ADP, serotonin, and thromboxane A2 (TXA2).^[45]^ These platelets can infiltrate the tumor microenvironment through focal adhesion kinase (FAK) activation, contributing to the progression of the tumor.^[45]^ Additionally, neutrophils can directly support tumor growth by secreting various chemokines and cytokines, and by recruiting other cells that promote tumor development to the tumor microenvironment.^[46]^ The presence of neutrophils in the tumor microenvironment is stimulated by vascular endothelial growth factor and interleukin 8 released by cancer cells, which in turn trigger the production of platelet-derived growth factor, fibroblast growth factor, matrix metalloproteinase, and interleukin 6. These elements play a crucial role in the initiation and progression of cancer.^[47]^ In contrast to platelets and neutrophils, lymphocytes operate as anti-tumor agents by triggering the p53 signaling pathway and secreting IL-17 to cause tumor cell death and stop tumor cell growth.^[48]^ Besides, persistent activation of T cells can enhance the anti-tumor effects of tumor-infiltrating T lymphocytes, leading to the apoptosis of cancer cells by presenting tumor-associated antigens to lymphocytes. This interaction is essential for enhancing adjunctive therapies and preventing tumor recurrence.^[49,50]^ Thus, high SII denotes high neutrophil and platelet counts, but low lymphocyte counts are often linked to tumor cell invasion and metastasis and result in poor survival. Likewise, the prognostic value of LMR, which reflects the immune status of the patient, is calculated from peripheral blood lymphocyte and monocyte count. Lymphocytes play a crucial anti-tumor role as described above. Furthermore, studies have shown that monocytes could promote tumor progression by secreting various cytokines, destroying extracellular matrix and promoting angiogenesis.^[51]^ Monocytes have the ability to transform into tumor-associated macrophages (TAMs) and dendritic cells within the tumor microenvironment (TME), supporting tumor growth and inhibiting the immune response.^[52]^ TAMs can stimulate angiogenesis by releasing growth factors and chemokines that aid in the progression of cancer.^[51]^ Therefore, high LMR is related to high levels of lymphocytes and low levels of monocyte, which are associated with good survival.

The limitations of this meta-analysis were numerous. First, insufficient numbers of studies evaluating SII and DFS were available and publication bias was existed in studies on LMR and pooled OS. As a result, the lack of statistical power may have reduced the dependability of the combined data. Additionally, most of the studies that were included in the analysis were carried out in Asia, which creates doubt about the generalizability of our results to other regions. Third, there were differences in the cutoff values amongst the investigations. The cutoffs in the included research didn’t reach a predetermined level, and few publications indicate how they were determined. Although we pooled the results according to SII > 200 and LMR > 4.21 in the subgroup analysis, bias may exist because using diverse cutoff values potentially leads to a different conclusion in the same article, which may influence the pooled results of this analysis. In the fourth place, the majority of the articles included in the study were retrospective in nature rather than RCTs, thereby diminishing their persuasiveness. Retrospective studies are more prone to biases such as lost to follow-up and information bias. In addition, some adjuvant drugs such as steroids which could affect overall survival in patients with oral cavity cancer^[53]^ may be used in the course of cancer. Furthermore, because people of different ages have different health conditions, age may also affect the prognosis of cancer and lead to selection bias in included studies. Moreover, factors like the levels of platelets, neutrophils, and lymphocytes in patients can be influenced by their condition, infection status, and other related variables. These confounding factors were not sufficiently accounted for in the initial articles.

In summary, our results showed that pretreatment SII and LMR represent independent predictive factors for OS and DFS, and that SII and LMR may be effective prognostic biomarkers in patients with oral cavity cancer.

## Author contributions

**Data curation:** Jingbo Yang.

**Project administration:** Yifeng Xing.

**Supervision:** Xiaojie Xing.

**Writing – original draft:** Jianghan Xu.

**Writing – review & editing:** Yanjun Lin.
